# Risk factors for an infection with *Coxiella burnetii* in German sheep flocks

**DOI:** 10.1017/S0950268820002447

**Published:** 2020-10-14

**Authors:** A. Wolf, T. L. Prüfer, C. Schoneberg, A. Campe, M. Runge, M. Ganter, B. U. Bauer

**Affiliations:** 1 Clinic for Swine, Small Ruminants and Forensic Medicine, University of Veterinary Medicine Hannover, Foundation, Hannover, Germany; 2 Lower Saxony State Office for Consumer Protection and Food Safety (LAVES), Food and Veterinary Institute Braunschweig/Hannover, Hannover, Germany; 3 Department of Biometry, Epidemiology and Information Processing (IBEI), WHO Collaborating Centre for Research and Training for Health at the Human-Animal-Environment Interface, University of Veterinary Medicine Hannover, Foundation, Hannover, Germany

**Keywords:** *Coxiella burnetii*, lambing all year-round, logistic regression, sheep, questionnaire

## Abstract

In Germany, sheep are the main source of human Q fever epidemics, but data on *Coxiella burnetii* (*C. burnetii*) infections and related risk factors in the German sheep population remain scarce. In this cross-sectional study, a standardised interview was conducted across 71 exclusively sheep as well as mixed (sheep and goat) farms to identify animal and herd level risk factors associated with the detection of *C. burnetii* antibodies or pathogen-specific gene fragments via univariable and multivariable logistic regression analysis. Serum samples and genital swabs from adult males and females of 3367 small ruminants from 71 farms were collected and analysed using ELISA and qPCR, respectively. On animal level, univariable analysis identified young animals (<2 years of age; odds ratio (OR) 0.33; 95% confidence interval (CI) 0.13–0.83) to reduce the risk for seropositivity significantly (*p* < 0.05). The final multivariable logistic models identified lambing all year-round (OR 3.46/3.65; 95% CI 0.80–15.06/0.41–32.06) and purchases of sheep and goats (OR 13.61/22.99; 95% CI 2.86–64.64/2.21–239.42) as risk factors on herd level for *C. burnetii* infection detected via ELISA and qPCR, respectively.

## Introduction

Q fever is an infectious zoonotic disease caused by the obligate intracellular and Gram-negative bacterium *Coxiella (C.) burnetii.* Domestic ruminants are traced as its most common reservoir and are widely recognised as the main source for human infections [1]. Clinical manifestation in ruminants may vary from asymptomatic infection to abortion, premature delivery, stillbirth and weak offspring [2]. Infected ruminants shed the pathogen through birth products, milk, faeces and urine [3]. In the environment, *C. burnetii* can survive in a highly resilient spore-like form [1]. Transmission by bacterial contaminated aerosols or dust is the most common route of human infection and a radius of 5 km around infected farms identified as being exposed to highest risk [1, 4]. Dry and windy weather conditions favour the spread of the pathogen [5]. Moreover, ticks are also considered to be involved in the infection cycle of *C. burnetii* in sheep [6].

In several European countries, e.g. Bulgaria and the Netherlands, dairy goats were held as responsible for sizeable human Q fever epidemics [7, 8]. In contrast, lambing sheep were identified as a primary source of human *C. burnetii* epidemics in Germany, where a number of small-scale outbreaks occurred within the last two decades with a maximum of 331 infected individuals in one of the outbreaks [9]. A recently conducted study revealed a herd prevalence of 26–36.6%; 13.9% of *C. burnetii*-positive sheep flocks in Germany detected using ELISA and qPCR, respectively [10].

In humans, the source for *C. burnetii* infections can usually be detected in retrospective studies [11]. The high tenacity of *C. burnetii*, the variation or even the absence of clinical signs in sheep and the lack of many aspects of the pathogen's epidemiology makes it particularly difficult to identify its way of introduction into the small ruminant population. However, several risk factors were identified for sheep and sheep flocks. For example, larger herds [12–15] and more breeding ewes within a flock [12, 15, 16] increased the chance for seropositivity. Furthermore, the seroprevalence and the risk for the detection of *C. burnetii* antibodies were more prevalent in older animals (>1–2 years) [15, 17, 18] and females having already given birth in contrast with nulliparous replacement animals [15]. Contact with other flocks [15], one or several supply addresses for ewes [19] or returning loaned sheep [16] also increased the risk of infection determined by the detection of antibodies. According to some reports, the likelihood of seropositive sheep increased with the number of goats within a radius of 10 km [12, 19]. However, Meadows *et al*. [16] reported no significant influence of goats on the *C. burnetii* status of sheep. Also, reproductive disorders such as infertility during the previous year [15] and more than six stillborn lambs in the subsequent lambing season [19] were associated with seropositivity.

Although sheep play an important role with regards to human infections in Germany [9], there remains a need for reliable data on risk factors for sheep exposure to *C. burnetii*. Consequently, the purpose of the present study is to identify risk factors for a *C. burnetii* infection in sheep flocks on individual animal and herd level in five federal states in Germany previously tested using ELISA and qPCR, respectively [10].

## Material and methods

### Study area

In relation to cattle or swine populations, the number of small ruminants in Germany is comparatively small. According to the German Federal Statistical Office via their GENESIS-Online Database, Germany counted 19556 sheep farms with approximately 1.83 million sheep in 2016 [20]. The vast majority of German farms (71.7%) shelter fewer than 50 sheep, while only 5.1% count over 500 [21]. Most flocks are run by hobby farmers, while professional farmers keeping more than 500 sheep – though less frequent – account for the majority of reproducing sheep. Numbers of farms and sheep in each federal state vary substantially and management approaches in sheep farming vary mainly between northern and southern Germany. Besides the sedentary husbandry system, traditional transhumance (migrating flocks) is still practised, especially in the southern federal states of Baden-Wuerttemberg (BW) and Bavaria (BAV). In this part of Germany, goats are frequently used to manage scrub in protected natural areas. Contrastingly, in northern federal states, especially Schleswig-Holstein (SH), sheep are used particularly for coastal protection on dikes while goats are difficult to keep under such conditions. As a consequence, there is a larger share of mixed (sheep and goat) farms in the southern federal states (BAV: 19.9%; BW: 30%) than in northern Germany (SH: 11.8%; Lower Saxony LS: 14%; North Rhine-Westphalia NRW: 14.4%) [20, 22]. In Germany, various sheep breeds are kept with a focus on meat sheep breeds with spring lambing in northern parts, while southern federal states mainly focus on Merino breeds with year-round, i.e. aseasonal lambing. These differences in husbandry impact variabilities of herd structures with regards to age and gender distribution. Nevertheless, female sheep (⩾1 year of age and mated females younger than 1 year) make up the largest part of the flock (64.4%), followed by lambs (<1 year of age, 32.6%) and sires, muttons and other sheep (3%) [20].

In the present study, we cover the five federal states of Germany displaying the highest number of farms/sheep: SH (1580/205685), LS (2167/197718), NRW (2238/159409), BW (2716/243558) and BAV (5140/317507). These states represent 70.8% of farms and 61.3% of the sheep population in Germany [20].

### Study design and detection methods

The current risk factor analysis of a *C. burnetii* infection is based on data taken from a recently published prevalence study. Details of farm and animal selection, sampling procedure and laboratory tests are published elsewhere [10].

In total, 3367 animals from 71 farms across five federal states were sampled and analysed using ELISA and qPCR, respectively. For each sampled animal, individual ear tag number, species (sheep or goat), sex, age and reproductive status of females (gimmer or ewe) were recorded for subsequent analysis of animal level risk factors for *C. burnetii* infection. In addition, a standardised interview was conducted by either author (AW or BUB) with the farm's manager on the animal sampling day to ascertain herd level risk factors. The standardised questionnaire consisted of questions concerning: (1) general farm indicators, (2) information on livestock kept on the farm, (3) husbandry system, (4) flock history, (5) diseases of humans living or working on the farm, (6) last lambing season and (7) current mating season. Before the first visit, the questionnaire was tested in three farms not included in the study. Variables of the sample list and the questionnaire with hypothetical high relevance were selected to identify risk factors at animal and herd level. Furthermore, mean humidity and temperature during sampling were retrieved from meteorological stations from the German weather service closest to the farms.

### Statistical analysis

#### Correlation analysis

Due to the large number of possible risk factors, we first verified that all variables differ from one another in terms of content. A correlation analysis was carried out to support this step. For this purpose, the following measures were determined and confirmed for correlation: Cramer's *V* > 0.5 for qualitative variables, ANOVA (equal variances) or Kruskal–Wallis test (unequal variances) *P* > 0.05 and coefficient of determination *R*^2^ > 0.1 for qualitative and quantitative variables and Pearson correlation coefficient > 0.7 for quantitative variables. Either correlated variables were summarised, one of them removed for further analysis but considered in the subsequent interpretation of the results or, if there was a moderate correlation, both variables were included in the model selection using an interaction term.

#### Risk factor analysis

A risk factor analysis was conducted to identify risk factors for an infection with *C. burnetii* at herd and animal level. The target variables (ELISA and qPCR) were dichotomised (positive/negative). Moreover, the geographical location was dichotomised (North = SH, LS, NRW; South = BAV, BW) to reduce results’ distortion. Due to the deviating infection rates and the different farm management systems in these two regions [10], the geographical location of the examined farms was considered as a confounder and therefore the model was stratified for the two regions.

For the risk factors at herd level, univariable and multivariable logistic regression models were provided for ELISA and qPCR results, respectively (PROC LOGISTIC, SAS, Institute Inc., Cary, NC, USA). For risk factor analysis at animal level, the farm was considered as a cluster variable. To do this, we took an extended generalised linear model approach to take the hierarchical structure of the data into account (PROC GENMODE, SAS, Institute Inc.). The parameters were estimated by using generalised estimating equations [23].

Odds ratio (OR), a 95% confidence interval (CI), Akaike Information Criterion (AIC) at herd level or Quasilikelihood under the Independence Model Criterion (QIC) at animal level and *p* values were calculated for categorical and continuous variables. A variable was used for further analysis if it had a *p* value lower than 20% (*p* < 0.20) of the model [24]. Moreover, a distinctive OR < 0.75 or OR > 1.33, and a reasonable corresponding 95% CI (ICI > 0.001; uCI < 999.99) led to the variable being taken further into account. In rare cases, a variable took on the same value on all observed farms. As a result, it was impossible to calculate meaningful ORs and CIs and the corresponding variables were not considered for the multivariable models. These criteria allowed variables to be considered for further analysis if they did not have a *p* value lower than 20% but still had a distinctive OR. Thus, the multivariable model could be selected from the largest number of possible risk factors and the probability of wrongly removing influencing factors was minimised.

Hereafter, we carried out a forward selection with the variables that met the abovementioned criteria. The variables which most improved the model fittings and whose addition achieved the best *p* values of the models were selected. The addition of the variables to the models was terminated either if all variables were included, or if the addition of variables led to no further improvement of the model fittings and the *p* values. In the ultimate step, the final models were each examined for collinearity using the variance inflation factor.

## Results

### Univariable analysis

#### Risk factors on animal level for an infection with *C. burnetii* detected using ELISA or qPCR

The animals’ age was a significant (*p* < 0.05) risk factor for an infection with *C. burnetii* detected using ELISA (Table 1). The likelihood for the detection of antibodies was reduced by two-thirds in animals younger than 2 years of age. None of the variables on animal level (sex, species and age) had any significant influence on the detection of *C. burnetii* by qPCR (Table 2). Tables 1 and 2 also show the apparent prevalence at an individual animal level of sex, species and age. Because of non-evaluable results using ELISA and especially qPCR and occasionally missing age indication, not all of the 3367 sampled animals could be included.

**Table 1.** Univariable logistic regression analysis of risk factors on animal level with farm considered as cluster variable for an infection with *Coxiella burnetii* detected using ELISA in 71 sheep flocks in Germany (2017/2018)

Variable	Category	Apparent prevalence of positive animals/animals total (%)	Odds ratio (OR)	95% confidence interval (CI)	*p* value
Sex	Male	6/449 (1.34)	1.74	0.81–3.75	0.15
	Female	75/2899 (2.59)			
Species	Goat	16/447 (3.58)	0.70	0.21–2.28	0.55
	Sheep	65/2901 (2.24)			
Age	⩾2 years	76/2896 (2.62)	0.33	0.13–0.83	0.02
	<2 years	5/386 (1.30)

**Table 2.** Univariable logistic regression analysis of risk factors on animal level with farm considered as cluster variable for an infection with *Coxiella burnetii* detected using qPCR in 71 sheep flocks in Germany (2017/2018)

Variable	Category	Apparent prevalence of positive animals/animals total (%)	Odds ratio (OR)	95% confidence interval (CI)	*p* value
Sex	Male	11/380 (2.89)	0.53	0.25–1.10	0.09
	Female	65/2606 (2.49)			
Species	Goat	25/397 (6.30)	0.97	0.48–1.94	0.93
	Sheep	51/2589 (1.97)			
Age	⩾2 years	71/2603 (2.73)	0.63	0.25–1.62	0.34
	<2 years	5/343 (1.46)			

#### Risk factors on herd level for an infection with *C. burnetii* detected using ELISA

The categorical variables, infestation with ticks, lambing on pasture and purchases of sheep and goats had a significant influence with the detection of *C. burnetii* infections using ELISA (Table 3). All farms stated their purchases (breeding sires/females or sires and females/no purchases) within the last 12 months in the questionnaire. On the whole, purchased animals were introduced into the flock without quarantine. The majority of farms purchased exclusively breeding sires (*n* = 41), followed by 19 farms without any purchases. The remaining 11 farms either bought breeding sires and females (*n* = 8) or only breeding females (*n* = 3). The proportion of seropositive farms (positive farms/examined farms) was 29.3% (12/41) for exclusively buying breeding sires, 10.5% (2/19) for no purchases and 54.5% (6/11) for purchases with females or sires with females.

**Table 3.** Univariable logistic regression analysis of risk factors on herd level for an infection with *Coxiella burnetii* detected using ELISA in 71 sheep flocks in Germany (2017/2018)

Variable	Category	Apparent prevalence of positive farms/farms total (%)	Odds ratio (OR)	95% confidence interval (CI)	*p* value
Categorical variables					
Swine	No	19/64 (29.69)	0.34	0.04–3.15	0.30
	Yes	1/7 (14.29)			
Cattle	No	13/46 (28.26)	1.05	0.34–3.22	0.93
	Yes	7/25 (28)			
Poultry	No	11/37 (29.73)	0.73	0.25–2.15	0.56
	Yes	9/34 (26.47)			
Dogs	No	1/3 (33.33)	0.96	0.08–12.04	0.98
	Yes	19/68 (27.94)			
Cats	No	8/32 (25)	1.13	0.38–3.37	0.82
	Yes	12/39 (30.77)			
Wild birds	No	1/6 (16.67)	1.63	0.17–15.84	0.66
	Yes	19/65 (29.23)			
Game	No	0/3 (0)	>999.99	<0.001->999.99	0.11
	Yes	20/68 (29.41)			
Rodent control	No	2/10 (20)	1.71	0.32–9.26	0.52
	Yes	18/61 (29.51)			
Infestation with ticks	No	0/15 (0)	>999.99	<0.001->999.99	**0.003**
	Yes	20/56 (35.71)			
Ectoparasitic treatment	No	8/27 (29.63)	0.99	0.33–2.97	0.98
	Yes	12/44 (27.27)			
Lambing behaviour	Spring	8/47 (17.02)	2.62	0.75–9.13	0.13
	Aseasonal	12/24 (50)			
(Main)Time of lambing	Spring	6/18 (33.33)			0.30
	Summer Autumn Winter	4/8 (50) 2/5 (40) 8/40 (20)	0.94 0.44 0.31	0.14–6.15 0.05–4.02 0.08–1.27	
Lambing location	Stable	8/45 (17.78)	6.61	1.36–32.25	**0.01**
	Pasture	12/26 (46.15)			
Husbandry system	Sedentary	16/64 (25)	4.05	0.76–21.71	0.10
	Migrating	4/7 (57.14)			
Participation on animal market	No	5/24 (20.83)	1.39	0.42–4.64	0.59
	Yes	15/47 (31.91)			
Purchases	No	2/19 (10.53)	5.88	1.85–18.73	**0.002**
	Yes	18/52 (34.62)			
Continuous variables					
Herd size	1 animal increase	–	1.000	0.999–1.001	0.53
	10 animals increase	–	1.003		
	100 animals increase	–	1.027		
	1000 animal increase	–	1.307		
Breeding sires/females	0.01 (1/100)	–	1.002		
	0.1 (10/100)	–	1.025		
	1 (100/100)	–	1.277	<0.001–>999.99	0.98
Humidity	1% increase	–	1.027	0.97–1.09	0.34
	10% increase	–	1.306		
	25% increase	–	1.949		
Temperature	1 °C increase	–	0.984	0.85–1.13	0.82
	5 °C increase	–	0.923		
	10 °C increase	–	0.852		

Significant variables (*p* < 0.05, likelihood ratio test) are printed in bold.

Although insignificant, the categorical variables of wild birds (common ravens, pigeons, sparrows, wild geese), game (wild boars, mouflons, red, fallow, roe and sika deer and foxes), rodent control, aseasonal lambing, migrating flocks and participation on animal markets were linked to an increased likelihood of antibody detection, while the OR of the variables, presence of swine and poultry on the farm and lambing either in autumn or winter hinted to a lower risk of antibody detection in the present study. Other variables such as the presence of cattle, cats or dogs, ectoparasitic treatment and summer lambing did not appear to influence the serological findings. Furthermore, none of the continuous variables had a significant impact on the detection of *C. burnetii* antibodies in German sheep flocks, although the OR indicated that an increasing mean humidity leads to a heightened seropositivity.

#### Risk factors on herd level for an infection with *C. burnetii* detected using qPCR

The categorical variables poultry and purchases of sheep and goats had a significantly positive influence on the detection of a *C. burnetii* infection using qPCR (Table 4). The proportion of farms (positive farms/examined farms) was 12.2% (5/41), 5.3% (1/19) and 36.4% (4/11) for purchases of exclusively breeding sires, no purchases and buying only females as well as females and sires, respectively.

**Table 4.** Univariable logistic regression analysis of risk factors on herd level for an infection with *Coxiella burnetii* detected using qPCR in 71 sheep flocks in Germany (2017/2018)

Variable	Category	Apparent prevalence of positive farms/farms total (%)	Odds ratio (OR)	95% confidence interval (CI)	*p* value
Categorical variables					
Swine	No	9/64 (14.06)	0.89	0.09–8.92	0.92
	Yes	1/7 (14.29)			
Cattle	No	7/46 (15.22)	0.81	0.18–3.64	0.78
	Yes	3/25 (12)			
Poultry	No	2/37 (5.41)	4.97	0.94–26.44	**0.04**
	Yes	8/34 (23.53)			
Dogs	No	1/3 (33.33)	0.37	0.03–5.29	0.48
	Yes	9/68 (13.24)			
Cats	No	3/32 (9.38)	1.74	0.39–7.76	0.46
	Yes	7/39 (17.95)			
Wild birds	No	1/6 (16.67)	0.50	0.04–5.91	0.59
	Yes	9/65 (13.85)			
Game	No	1/3 (33.33)	0.37	0.03–5.29	0.48
	Yes	9/68 (13.24)			
Rodent control	No	0/10 (0)	>999.99	<0.001->999.99	0.27
	Yes	10/61 (16.39)			
Infestation with ticks	No	1/15 (6.67)	1.46	0.15–13.94	0.73
	Yes	9/56 (16.07)			
Ectoparasitic treatment	No	4/27 (14.81)	1.05	0.25–4.35	0.95
	Yes	6/44 (13.64)			
Lambing behaviour	Spring	3/47 (6.38)	2.38	0.48–11.73	0.27
	Aseasonal	7/24 (29.17)			
(Main)Time of lambing	Spring	2/18 (11.11)			0.81
	Summer Autumn Winter	1/8 (12.5) 2/5 (40) 5/40 (12.5)	0.45 1.50 0.67	0.03–6.61 0.13–17.25 0.10–4.42	
Lambing location	Stable	7/45 (15.56)	1.52	0.27–8.56	0.63
	Pasture	3/26 (11.54)			
Husbandry system	Sedentary	9/64 (14.06)	0.89	0.09–8.92	0.92
	Migrating	1/7 (14.29)			
Participation on animal market	No	2/24 (8.33)	1.60	0.29–8.74	0.58
	Yes	8/47 (17.02)			
Purchases	No	1/19 (5.26)	9.43	1.75–50.94	**0.004**
	Yes	9/52 (17.31)			
Continuous variables					
Herd size	1 animal increase	–	1.000	0.998–1.001	0.49
	10 animals increase	–	0.996		
	100 animal increase	–	0.956		
	1000 animal increase	–	0.637		
Breeding male/female	0.01 (1/100)	–	1.115		
	0.1 (10/100)	–	2.975		
	1 (100/100)	–	>999.99	<0.001–>999.99	0.49
Mean humidity	1% increase	–	0.932	0.869–1	**0.04**
	10% increase	–	0.497		
	25% increase	–	0.174		
Temperature	1° C increase	–	1.112	0.944–1.31	0.21
	5° C increase	–	1.702		
	10° C increase	–	2.897		

Significant variables (*p* < 0.05, likelihood ratio test) are printed in bold.

Most categorical variables did not have a significant impact on an infection with the pathogen detected by qPCR as using ELISA. Nevertheless, the OR of the variables presence of cats on the farm, infestation with ticks, rodent control, aseasonal lambing, lambing in autumn, on-pasture lambing and the participation on animal markets suggested an increased risk of *C. burnetii* infections detected by qPCR. The presence of dogs, wild birds, game and lambing either in summer or winter lessened the incidence of *C. burnetii* infection at herd level in the present study. The presence of swine and cattle, ectoparasitic treatment and husbandry system did not have any impact based on our data. Among the continuous variables, pathogen detection depended significantly on the humidity. An increase in the mean humidity resulted in a decrease in detection via qPCR. Although insignificant, an increase in temperature increased the risk for a *C. burnetii* infection. Moreover, a significant increase in the herd size decreased the probability of detecting the pathogen via qPCR, while the increase in the ratio of breeding sires to females in a flock tended to amplify the risk of a *C. burnetii* infection. Both continuous variables were not significant.

### Multivariable analysis

#### Risk factors on animal level for an infection with *C. burnetii* detected using ELISA or qPCR

As expanding the model did not achieve an improvement of the model fittings, no multivariable analyses were carried out.

#### Risk factors on herd level for an infection with *C. burnetii* detected using ELISA

The final multivariable model contained the variables lambing behaviour, purchases and poultry kept on the farm (Table 5).

**Table 5.** Multivariable logistic regression analysis of risk factors on herd level for an infection with *C. burnetii* detected using ELISA in 71 sheep flocks in Germany (2017/2018)

Variable	Category	Odds ratio (OR)	95% confidence interval (CI)	*p* value
Poultry	No	0.21	0.04–1.01	0.05
	Yes			
Lambing behaviour	Spring	3.46	0.80–15.06	0.10
	Aseasonal			
Purchases	No	13.61	2.86–64.64	0.001
	Yes			

The final model had an Akaike Information Criterion (AIC) of 59.54 and a *p* value (likelihood ratio test) of 0.001.

#### Risk factors on herd level for an infection with *C. burnetii* detected using qPCR

Four *C. burnetii* infection-associated risk factors were included in the final model: purchases, lambing behaviour, presence of game and mean humidity (Table 6).

**Table 6.** Multivariable logistic regression analysis of risk factors on herd level for an infection with *C. burnetii* detected using qPCR in 71 sheep flocks in Germany (2017/2018)

Variable	Category	Odds ratio (OR)	95% confidence interval (CI)	*p* value
Game	No	0.03	<0.001–1.60	0.08
	Yes			
Lambing behaviour	Spring	3.65	0.41–32.06	0.24
	Aseasonal			
Purchases	No	22.99	2.21–239.42	0.01
	Yes			
Mean humidity	1% increase	0.91	0.83–1	0.06

The final model had an Akaike Information Criterion (AIC) of 34.83 and a *p* value (likelihood ratio test) of 0.002.

## Discussion

Some of the variables obtained from the questionnaire and the sample list were identified as being associated with an increased *C. burnetii* infection hazard. However, the data gathered from the questionnaire were included in the study for prevalence estimation in 71 German sheep flocks [10] and no sample calculations were undertaken for subsequent risk factor analysis. The lack of significant impact of some risk factors could be due to the limited numbers of farms and the huge variability in farm management and husbandry. Therefore, the generated risk factors are inconclusive, but the results provide an indication of possible influences on an infection with *C. burnetii* detected using ELISA and qPCR, respectively [25]. Further research is needed to confirm the results in this study.

In general, the risk factor analysis was based on the results of two different methods to detect an infection of *C. burnetii* in sheep and goats. The applied ELISA was based on the detection of IgG Phase I and Phase II antibodies and rendered no information about time and progress of an infection. Additionally, according to other studies, some small ruminants did not seroconvert although they were shedding *C. burnetii* and *vice versa* [26–28]. Moreover, antibodies were described to last for several months after an acute infection without the presence of the pathogen [27]. In contrast, the detection of *C. burnetii*-DNA by qPCR showed that the pathogen was circulating within the flock, which indicates a recent infection as long as chronic shedders have not yet been reported in small ruminants. These circumstances could explain some opposing findings of risk factor analysis in the present study.

### Risk factors on animal level for an infection with *C. burnetii*

Age was identified as the only significant risk factor based on the ELISA results at animal level. Animals 2 years of age or older presented a higher seroprevalence. This is in line with studies performed in other European countries [15, 17, 18]. Adults were more likely to form antibodies due to a higher chance of getting in contact with the pathogen during their lifetime [15, 17]. Moreover, the proportion of infected ewes (2.95/2.92%) in this study was higher in comparison to gimmers (young female before first lambing) (1.34/0.91%) detected using ELISA and PCR, respectively. This is in accordance with García-Pérez *et al*. [18] and Rizzo *et al*. [15], who reported a lower prevalence in replacement animals not exposed to the pathogen until lambing.

Although not statistically significant, the results of the presented regression analysis suggest that goats within a sheep flock have a higher probability to contract an infection with *C. burnetii.* Overall, the detection rate of the pathogen was higher in goats compared to sheep regardless of the applied test system, although the proportion of goats in the present study is overrepresented and should be accounted for cautiously when interpreting the results. A similar observation was made in a study conducted with small ruminants with 25.7% seropositive goats compared to 16.3% seropositive sheep in mixed flocks [15]. Interestingly, in the same study, no significant difference in seroprevalence was detected between sheep (11.42%) and goats (10.34%) originating from pure sheep and pure goat farms. Moreover, a higher goat density within a 10 km radius was identified as a risk factor for a *C. burnetii* infection in sheep [12, 19]. Therefore, the cross-species interaction of *C. burnetii* between sheep and goats needs further investigation to identify possibly species-specific characteristics.

### Risk factors on herd level for an infection with *C. burnetii*

The univariable analysis identified infestation with ticks as a significant risk factor for a *C. burnetii* infection based on antibody detection at herd level. Ticks were described as vectors for *C. burnetii* [1, 6]. However, the pathogen was scarcely detected in ticks in Germany [29, 30]. Recently, however, both common tick species (*Dermacentor marginatus* and *Ixodes ricinus*) were experimentally infected with *C. burnetii* and remarkable amounts of the pathogen were found to be shed with their faeces [31]. Therefore, transmission very likely occurs predominantly via tick faeces. The relevance of the particular tick species remains unclear. Further research to verify the transmission of the pathogen from ticks to livestock is needed. In this context, it is worth mentioning that ectoparasitic treatment had no significant influence on the infection with *C. burnetii* in our analysis.

The risk of a positive test outcome from ELISA in the current study for flocks with on-pasture lambing was significantly higher than for flocks lambing indoors. This is in line with findings from Schimmer *et al.* on individual sheep level [19]. Additionally, lambing on pasture increased the risk for an infection detected by qPCR, although statistically insignificantly. On the one hand, outdoor lambing was suspected to reduce the exposure of sheep to *C. burnetii* [16] possibly due to a lower infection pressure in comparison to lambing in the stable. On the other hand, implementation of hygienic measures (disinfection of lamb pens, disposal of afterbirth) is probably rarer on pasture and pathogen containing material could thus be spread by wind and contaminate the grazing area. In addition, the frequency of grazing on contaminated pastures might have an influence on the risk of infection. In flocks with aseasonal lambing, it is likely that only a small part of all the available grazing ground is appropriate for a mob of lambing ewes. Therefore, these specific pastures – often located nearby the farmhouse – are frequently used by flocks with lambing all year-round. Moreover, getting into contact with other flocks was identified as a risk factor and was more likely with small ruminants kept on pasture than housed animals year-round [15]. But contrary to the findings of Rizzo *et al*. [15], in the present study, besides poultry, neither other livestock, nor pets or game had a significant influence on the infection with *C. burnetii* at herd level.

The presence of poultry on a farm increased the risk for a *C. burnetii* infection detected by PCR significantly. Based on ELISA detection, poultry decreased the risk. The role of poultry as a reservoir for *C. burnetii* was summarised by Lang [32]. One should note that recent studies are rare. Antibodies against *C. burnetii* but no pathogen-specific DNA were detected in chicken [33]. Therefore, the influence of poultry as host for *C. burnetii* remains inconclusive.

In this study, aseasonal lambing was identified by multivariable analysis as a risk factor regardless of the detection method. Lambing all year-round might lead to constant circulation of the pathogen within the flock and a steady source of (renewed) infection for animals and humans alike. Merinos are an aseasonal sheep breed and thus lambing year-round. They are mainly kept in southern Germany. This may explain the higher occurrence of *C. burnetii* in the small ruminant population in this part of Germany and the frequently small-scale human epidemics especially in BW [9, 10].

Both logistic regression analyses identified purchases of sheep and goats to be significantly associated with a *C. burnetii* infection detected using ELISA or qPCR, respectively. Schimmer *et al*. [19] made a similar observation. They determined one or more supply addresses for ewes as a risk factor in sheep flocks in the Netherlands. In the present study, farms purchasing females tested positive more frequently than farms exclusively purchasing males. However, most farmers bought exclusively breeding sires, which reflects a common management practice in the German sheep industry. Buying females, ewes and gimmers, remains rare. Therefore, buying new breeding sires may increase the risk for *C. burnetii* infection. Venereal transmission of *C. burnetii* is not yet detailed conclusively, but detection of the pathogen in ram semen [34] and on preputial mucosa [10] was already demonstrated. This is supported by the observations made in the current study. The chance of finding *C. burnetii* by qPCR was higher in preputial than in vaginal swabs. On the other hand, the chance of being seropositive was higher in females. Females remain in a flock for years and thus have a higher opportunity to become infected during their lifetime [15, 17], whereas sires are exchanged after one or two mating seasons. This could explain the higher proportion of seropositive females. However, sex did not have a significant influence on the detection of a *C. burnetii* infection using ELISA and qPCR, respectively. Overall, animal movements pose a particularly high risk for the entry of *C. burnetii* into the flock [16, 19], especially when purchasing animals from different farms with unknown infection status.

An increasing mean humidity significantly reduced the risk for an infection detected by qPCR and an increasing temperature tended to increase the risk of shedding the pathogen. Different lambing seasons may be connected with the detected influence of the climate on the risk of infection. For instance, higher temperatures and lower relative humidity during summer lambing in the federal states BW and BAV and their continental climate may increase the risk for shedding and transmitting the pathogen. In the federal state of SH, experiencing a more maritime climate due to the location between North Sea and Baltic Sea, lower temperatures in combination with higher relative humidity during the normal lambing season in February and March may reduce the risk of an infection detected by qPCR. Environmental weather conditions and their influence on an infection with *C. burnetii* were investigated in some studies. Nusinovici *et al*. [35] suggested low precipitation and high temperature as a risk factor for an infection with the pathogen. In addition, van der Hoek *et al*. [36] described areas that favour the formation of dust constitutes a higher risk for human infection. Conversely, rain seemed to reduce transmission [37]. Therefore, high precipitation and high humidity may create worse conditions for the transmission and maintenance of the pathogen, while dry weather and wind blowing indicate an increase in the spread of *C. burnetii* [5].

In summary, age had a significant influence on the detection of *C. burnetii* antibodies at animal level. Older animals (⩾2 years of age) were more frequently seropositive than younger ones. Therefore, the composition of the flock, especially the replacement rate might have an influence on transmission and circulation of the pathogen in the flock. The multivariable analysis identified purchases and lambing all year-round as risk factors for a *C. burnetii* infection at herd level detected using ELISA and qPCR, respectively. The results and observations compiled in this study are of particular use in establishing an active monitoring and surveillance system for the German small ruminant population, which may contribute to prevent the transmission of *C. burnetii* to animals and humans alike.

## Data Availability

The data that support the findings of this study are available from the corresponding author.
